# Supplementary material for: Modulation of Resting Connectivity Between the Mesial Frontal Cortex and Basal Ganglia
Source: Front Neurol. 2019 Jun 5;10:587. doi: 10.3389/fneur.2019.00587 (PMC6593304; doi:10.3389/fneur.2019.00587)
Supplement: Supplementary file 1 [file Table_1.DOCX]

## Supplementary Methods

### Seed definitions

The anatomically defined ROIs which were manually created or altered using MarsBaR ROI toolbox [1] for SPM. The broad mesial prefrontal ROI was defined with the posterior border as the extent of the SMA and the anterior border as the anterior extent of the dorsal ACC. The statistics for positive and negative functional connectivity of the medial prefrontal cortex seed are reported in Supplementary Table S1.

The mid cingulate ROI had an anterior border of the posterior end of the genu of the corpus callosum and posterior border a vertical line through the anterior commissure - the same as the pre-SMA posterior border. The statistics for positive and negative functional connectivity of the mid-cingulate cortex seed are reported in Supplementary Table S2.

### Electrical field induced by H7 coil

The head, coils, and electric field were modeled with the electromagnetic finite element package MagNet (Infolytica, Inc., Canada). The electric field simulation methods were previously described in detail and validated experimentally [2]. Briefly, the human head was modeled as a homogeneous sphere with radius of 8.5 cm and isotropic conductivity of 0.33 S/m. The H7 coil consisted of two adjacent wings fixed at a relative angle of 90 degrees; each wings consisted of two layers of concentric elliptical windings with major axis ranging from 75–140 mm, and minor axis ranging from 70–125 mm; each layer has 4 turns. The coil windings were modeled as stranded copper wires with cross-sectional diameter of 4 mm. The electric field distribution was computed using the MagNet Time Harmonic solver, and scaled to match the output of a Magstim Rapid2 device. We estimated the electric field threshold for neural activation, *E*_th_, using a linear model of neuronal response to TMS [2].

### Supplementary Tables

|  | **p(FWE-corr)** | **K** | **T** | **Z** | **x** | **y** | **z** |
| --- | --- | --- | --- | --- | --- | --- | --- |
| **MPFC positive** |  |  |  |  |  |  |  |
|  |  |  |  |  |  |  |  |
| MPFC | <0.001 | 36108 | 36.7 | >8 | -10 | 19 | 61 |
|  |  |  | 35.73 | >8 | -10 | 31 | 56 |
|  |  |  | 33.56 | >8 | -1 | 7 | 70 |
| Cerebellum | <0.001 | 2608 | 16.49 | >8 | 29 | -79 | -32 |
|  |  |  | 14.27 | >8 | 36 | -62 | -28 |
|  |  |  | 11.2 | >8 | 34 | -60 | -53 |
| Cerebellum | <0.001 | 1245 | 12.24 | >8 | -27 | -74 | -30 |
|  |  |  | 10 | >8 | -34 | -65 | -25 |
|  |  |  | 5.52 | 5.26 | -24 | -58 | -23 |
| Lateral Parietal | <0.001 | 625 | 11.28 | >8 | 57 | -55 | 35 |
| Thalamus | <0.001 | 137 | 9.89 | >8 | -8 | -20 | 3 |
| Cerebellum | <0.001 | 24 | 7.44 | 6.86 | 6 | -55 | -42 |
|  |  |  | 5.87 | 5.57 | 6 | -60 | -49 |
| Midbrain | <0.001 | 34 | 7.02 | 6.53 | -1 | -23 | -30 |
| Cerebellum | <0.001 | 25 | 6.95 | 6.47 | -8 | -62 | -42 |
| Cerebellum | <0.001 | 46 | 6.6 | 6.18 | -1 | -53 | -9 |
| Cerebellum | <0.001 | 103 | 6.58 | 6.17 | -34 | -62 | -56 |
|  |  |  | 5.95 | 5.63 | -41 | -55 | -51 |
| Thalamus | <0.001 | 24 | 6.33 | 5.95 | 10 | -18 | 3 |
| Medial Parietal | <0.001 | 24 | 5.58 | 5.32 | -10 | -51 | 33 |
| Cerebellum | 0.011 | 4 | 5.4 | 5.15 | -24 | -39 | -28 |
|  |  |  |  |  |  |  |  |
| **MPFC negative** |  |  |  |  |  |  |  |
| Medial Parietal | <0.001 | 13212 | 14.78 | >8 | -17 | -60 | 21 |
|  |  |  | 14.47 | >8 | 15 | -51 | 19 |
|  |  |  | 13.75 | >8 | 15 | -67 | 28 |
| Lateral OFC | <0.001 | 136 | 9.66 | >8 | -27 | 35 | -14 |
| Lateral OFC | <0.001 | 106 | 9.52 | >8 | 22 | 33 | -16 |
| Cerebellum | <0.001 | 237 | 8.95 | >8 | 1 | -46 | -30 |
|  |  |  | 8.25 | 7.49 | -10 | -48 | -46 |
| Temporal | <0.001 | 150 | 8.23 | 7.48 | -50 | -60 | -2 |
| Cerebellum | <0.001 | 102 | 7.79 | 7.14 | 13 | -44 | -49 |
| Temporal | 0.001 | 18 | 6 | 5.68 | 48 | -51 | -4 |
| Cerebellum | 0.001 | 17 | 5.8 | 5.5 | -6 | -83 | -37 |
| Lateral Prefrontal | 0.001 | 19 | 5.49 | 5.24 | 41 | 33 | 19 |

###

**Supplementary Table S1.** Statistics for positive and negative functional connectivity of medial prefrontal cortex (MPFC) seed. Abbreviations: p(FWE-corr), whole brain (P<0.05) family-wise error corrected P value; K, cluster size; T, T statistic; Z, Z-score; xyz, peak voxel coordinates; OFC, Orbitofrontal Cortex.

|  | **p(FWE-corr)** | **K** | **T** | **Z** | **x** | **y** | **z** |
| --- | --- | --- | --- | --- | --- | --- | --- |
|  |  |  |  |  |  |  |  |
| **Mid Cingulate positive** |  |  |  |  |  |  |  |
| Mid Cingulate | <0.001 | 30314 | 54.16 | >8 | -6 | 14 | 35 |
|  |  |  | 52.52 | >8 | -3 | 7 | 40 |
|  |  |  | 51.1 | >8 | 3 | 17 | 35 |
| Dorsolateral PFC | <0.001 | 513 | 13.04 | >8 | 29 | 45 | 28 |
|  |  |  | 5.97 | 5.65 | 38 | 42 | 14 |
| Cerebellum | <0.001 | 300 | 10.92 | >8 | -31 | -53 | -51 |
|  |  |  | 6.14 | 5.8 | -17 | -62 | -53 |
| Temporal | <0.001 | 68 | 10.53 | >8 | -20 | -41 | 0 |
| Cerebellum | <0.001 | 233 | 10.11 | >8 | -34 | -53 | -28 |
|  |  |  | 6.94 | 6.46 | -43 | -58 | -30 |
| Temporal | <0.001 | 57 | 10.02 | >8 | 20 | -39 | 3 |
| Cerebellum | <0.001 | 356 | 9.9 | >8 | 34 | -51 | -51 |
|  |  |  | 7.61 | 6.99 | 20 | -48 | -56 |
|  |  |  | 7.05 | 6.55 | 13 | -60 | -51 |
| Cerebellum | <0.001 | 183 | 9.52 | >8 | 34 | -48 | -30 |
|  |  |  | 7.34 | 6.78 | 43 | -55 | -32 |
|  |  |  | 5.33 | 5.09 | 22 | -60 | -23 |
| Inferior Frontal | <0.001 | 90 | 7.69 | 7.06 | -29 | 35 | -14 |
| Occipital | <0.001 | 52 | 7.01 | 6.52 | -1 | -58 | -7 |
| Cerebellum | 0.001 | 20 | 6.42 | 6.03 | -3 | -34 | -42 |
| Occipital | 0.001 | 21 | 6.12 | 5.78 | -24 | -67 | 7 |
| Occipital | 0.011 | 4 | 5.26 | 5.04 | 52 | -60 | 3 |
| Occipital | 0.002 | 12 | 5.26 | 5.03 | 24 | -60 | 7 |
|  |  |  |  |  |  |  |  |
| **Mid Cingulate negative** |  |  |  |  |  |  |  |
| Cerebellum | <0.001 | 12058 | 12.82 | >8 | -13 | -74 | -30 |
|  |  |  | 11.61 | >8 | -31 | -74 | -32 |
|  |  |  | 11.31 | >8 | 41 | -62 | 59 |
| Temporal Cortex | <0.001 | 707 | 11.5 | >8 | 66 | -27 | -7 |
|  |  |  | 6.3 | 5.93 | 66 | -48 | -14 |
|  |  |  | 5.34 | 5.1 | 62 | -2 | -18 |
| Dorsolateral PFC | <0.001 | 494 | 11.17 | >8 | -43 | 10 | 56 |
|  |  |  | 6.79 | 6.34 | -57 | 21 | 21 |
| Dorsolateral PFC | <0.001 | 1278 | 10.83 | >8 | 48 | 14 | 52 |
|  |  |  | 10.42 | >8 | 52 | 21 | 45 |
|  |  |  | 7.43 | 6.85 | 43 | 17 | 19 |
| Cerebellum | <0.001 | 396 | 9.57 | >8 | -6 | -55 | -37 |
|  |  |  | 8.61 | 7.76 | -6 | -58 | -49 |
|  |  |  | 8.61 | 7.75 | 1 | -58 | -44 |
| Mid Cingulate | <0.001 | 301 | 8.31 | 7.53 | 3 | 12 | 12 |
|  |  |  | 8.25 | 7.49 | -6 | 14 | 12 |
|  |  |  | 7.9 | 7.22 | 6 | 3 | 17 |
| Temporal | <0.001 | 342 | 7.95 | 7.26 | -64 | -30 | -4 |
|  |  |  | 6.9 | 6.43 | -66 | -39 | -2 |
| Frontal Polar | <0.001 | 414 | 7.94 | 7.26 | 34 | 66 | 0 |
|  |  |  | 7.05 | 6.55 | 43 | 54 | -14 |
|  |  |  | 6.01 | 5.68 | 48 | 33 | -11 |
| Cerebellum | <0.001 | 157 | 7.91 | 7.23 | 8 | -37 | -16 |
|  |  |  | 6.91 | 6.44 | 17 | -27 | -30 |
|  |  |  | 6.6 | 6.18 | 17 | -14 | -30 |
| Inferior Frontal | <0.001 | 289 | 7.36 | 6.8 | -50 | 38 | -9 |
| Midbrain | <0.001 | 171 | 7.35 | 6.79 | -6 | -7 | -21 |
|  |  |  | 5.18 | 4.96 | 1 | 5 | -21 |
| Thalamus | <0.001 | 110 | 7.32 | 6.77 | 29 | -34 | 12 |
|  |  |  | 6.54 | 6.13 | 24 | -30 | 5 |
| Thalamus | <0.001 | 92 | 6.99 | 6.5 | -27 | -32 | 5 |
|  |  |  | 6.47 | 6.08 | -36 | -32 | 0 |
|  |  |  | 6.34 | 5.96 | -29 | -39 | 12 |
| Dorsomedial PFC | <0.001 | 111 | 6.93 | 6.45 | 1 | 40 | 45 |
| Cerebellum | <0.001 | 81 | 6.49 | 6.09 | -13 | -32 | -18 |
|  |  |  | 6.21 | 5.85 | -6 | -44 | -7 |
|  |  |  | 5.42 | 5.17 | -17 | -27 | -32 |
| Temporal | <0.001 | 93 | 6.22 | 5.86 | -59 | -11 | -21 |
|  | <0.001 | 29 | 6.03 | 5.7 | -22 | -20 | 31 |
| Cerebellum | 0.002 | 14 | 6.01 | 5.69 | 22 | -34 | -42 |
| Frontal | 0.001 | 15 | 5.92 | 5.61 | -20 | 26 | 12 |
| Medial Parietal | <0.001 | 27 | 5.82 | 5.52 | 3 | -74 | 54 |
| Posterior Cingulate | 0.003 | 11 | 5.79 | 5.5 | 1 | -51 | 7 |
| Frontal | 0.007 | 6 | 5.72 | 5.44 | -29 | 31 | 17 |
| Mid Cingulate | <0.001 | 40 | 5.66 | 5.38 | 17 | -14 | 26 |
|  |  |  | 5.46 | 5.21 | 10 | -16 | 21 |
|  |  |  | 5.31 | 5.08 | 15 | -30 | 21 |

**Supplementary Table S2.** Statistics for positive and negative functional connectivity of mid-cingulate cortex seed. Abbreviations: p(FWE-corr), whole brain (P<0.05) family-wise error corrected P value; K, cluster size; T, T statistic; Z, Z-score; xyz, peak voxel coordinates; PFC, prefrontal Cortex.

**Supplementary Figure S1.** The H7 helmet with the internal wiring and the spherical head model used for calculating the induced-electrical field decay with distance from the coil.

## Supplementary references

1. Brett M, A.J., Valabregue R, Poline JB (2002): Region of interest analysis using an SPM toolbox [abstract]. *Presented at the 8th International Conferance on Functional Mapping of the Human Brain* Available on CD-ROM in NeuroImage, Vol 16, No 2, abstract 497:

2. Deng, Z.D., S.H. Lisanby, and A.V. Peterchev (2011): Electric field strength and focality in electroconvulsive therapy and magnetic seizure therapy: a finite element simulation study. *J Neural Eng* 8: 016007.
