# Supplementary figures and images for: Modulation of Resting Connectivity Between the Mesial Frontal Cortex and Basal Ganglia
Source: Front Neurol. 2019 Jun 5;10:587. doi: 10.3389/fneur.2019.00587 (PMC6593304; doi:10.3389/fneur.2019.00587)

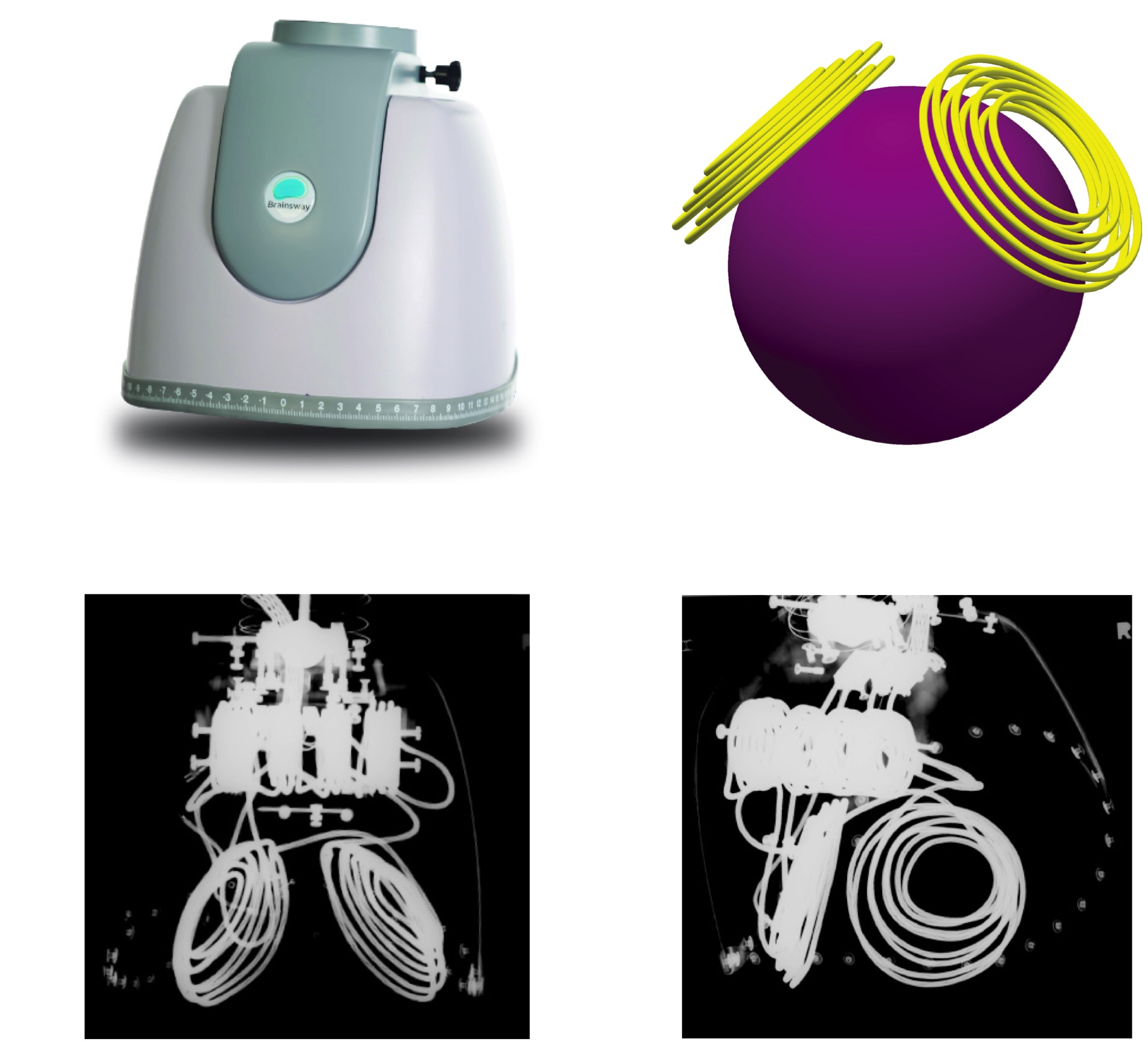

Supplement: Supplementary file 2 [file Image_1.JPEG]
